# The Effects of Day of the Week on Temporal Ambiguity Resolution

**DOI:** 10.1177/0033294120979686

**Published:** 2021-02-11

**Authors:** Srdan Medimorec

**Affiliations:** Department of Psychology, 5462 Teesside University, Middlesbrough, UK

**Keywords:** Temporal metaphor, temporal perspective, day of the week

## Abstract

Previous research has suggested that individuals’ resolution of temporal ambiguity relies on experiences in physical domains, as well as on future event valence and emotional experiences. In the current study, we investigate whether the interpretation of a temporally ambiguous question is modulated by the day of the week on which the study was conducted. We asked participants (*N* = 208) to resolve the ambiguous time question on different days of the week (Monday or Friday). The results of the experiment indicate differences in processing of temporal ambiguity between different days of the week. The study raises the interesting possibility that days of the week can have important implications for resolving temporal ambiguity.

## Introduction

Time is often described using spatial metaphors and this relation is well documented across many languages. Typically, future is conceptualized as being ahead, while past is behind relative to the self (Boroditsky & Ramscar, 2002; Lakoff & Johnson, 1980, 1999; McGlone & Harding, 1998). This future-front and past-back view of time is presented in English using two distinct perspectives: the moving ego (ME) and the moving time (MT) perspective (Lakoff & Johnson, 1980, 1999). In the ME perspective individuals think of themselves as moving forward in time relative to stationary future events (e.g., “We’re coming up on Christmas”), while in the MT perspective they think that future events are moving toward them (e.g., “Christmas is coming”; Boroditsky, 2000; McGlone & Harding, 1998).

Typically, the relation between temporal and spatial reasoning has been investigated using the temporally ambiguous question “*Next Wednesday’s meeting has been moved forward two days. What day is the meeting on now*?” (Boroditsky & Ramscar, 2002; Duffy & Evans, 2017; Margolies & Crawford, 2008). Clearly, one can interpret the question using either the ME or the MT perspective. Using the ME perspective, the response would be Friday, since the metaphor moves the event away into the future, while using the MT perspective the response would be Monday, indicating that the event is moving closer in time.

A critical question about the nature of the factors underlying the resolution of the ambiguous temporal question remains a matter of debate. Previous studies investigating this question have reported that attending to various extraneous spatial stimuli can elicit the use of a specific perspective of time (Boroditsky & Ramscar, 2002; Matlock et al., 2005; Ramscar et al., 2010). Boroditsky and Ramscar (2002) asked participants to either think of themselves as moving towards an object, or imagine the object moving towards them. Afterwards, participants answered the ambiguous temporal question. Individuals were more likely to adopt the ME perspective and answer Friday after thinking about themselves moving forward. If they thought about the object moving, they were more likely to adopt the MT perspective and answer Monday. In another demonstration of the phenomenon, individuals flying on a plane were more likely to adopt the ME perspective, while individuals waiting for a plane were more likely to adopt the MT perspective (Boroditsky & Ramscar, 2002). Even counting forward or backward elicited similar effects. Specifically, when individuals answered the ambiguous question after counting forward, they were more likely to adopt the ME perspective. After counting backward individuals were more likely to adopt the MT perspective (Matlock, 2010). The results of these studies were interpreted to indicate a relation between temporal ambiguity resolution and spatial reasoning.

On the other hand, there is evidence that an individual’s response to the “Wednesday’s meeting” question might be influenced by different extraneous non-spatial factors, such as event valence, emotional experiences, and personality traits (Duffy & Feist, 2014; Duffy et al., 2014; Margolies & Crawford; 2008; Richmond et al., 2012; Stocker, 2020). Duffy and Feist (2014) reported differences in temporal reasoning between introverts and extroverts. Introverts were more likely to adopt the MT perspective, while extroverts adopted the ME perspective, akin to individual differences between approach and avoidance. Richmond et al. (2012) reported that the participants in the anxiety-induced condition were more likely to judge that the event was approaching them (i.e., the MT perspective, indicated by more “Monday” responses). Taken together, these findings indicate that a range of factors may influence temporal ambiguity resolution.

In the current study we expand on previous research demonstrating the importance of contextual factors in the resolution of the “Wednesday’s meeting” question. Specifically, we investigate whether the answer to this question is modified by the day of the week on which the study was conducted. Ellis et al. (2015) have reported that individuals were fastest to retrieve the current day on Monday and Friday, suggesting that mental representations of Monday and Friday are more distinctive and richer compared to other days. By this account, Monday and Friday attract more associated concepts compared to other weekdays. Moreover, while Mondays are associated with more negative concepts, Fridays are associated with more positive concepts. In addition, there is evidence that mood tends to be more negative on Mondays and more positive on Fridays (Areni et al., 2011; Ellis et al., 2015; Stone et al., 2012). Thus, the mental and affective distinctiveness of Mondays and Fridays in conjunction with potential weekly mood changes and previous research demonstrating that various extraneous factors (e.g., affect) can influence the interpretation of the ambiguous question raise an interesting possibility of daily fluctuations (Monday vs. Friday) in the interpretation of the temporally ambiguous question.

We asked participants to resolve the ambiguous time question (i.e., the “Wednesday’s meeting” question) on different days of the week (Monday or Friday). Based on the reported salience effects of Mondays and Fridays, but also on the negative associations for Mondays and positive associations for Fridays, we expected that individuals would be more likely to adopt the MT perspective on Monday, and ME perspective on Friday.^1^

## Method

### Participants

A total of 231 participants were recruited via MTurk and SONA as part of a larger study. All participants were fluent English speakers located in the USA and received $4 in exchange for their participation. Participants were recruited on Mondays and Fridays during a two-week period (each participant was restricted to one sign-up). The final sample included 208 participants who completed the study (mean age = 32.41 (one preferred not to say), range = 18–74, female *n* = 87; Monday condition = 121). Exclusion criteria included failing to answer the ambiguous question or answering other than Monday or Friday.

### Procedure

After completing the consent form and demographic questionnaire, participants were asked the ambiguous temporal question (“*Next Wednesday’s meeting has been moved forward two days. What day is the meeting now that it has been rescheduled?”)*.^2^

## Results

The distribution of answers between the two study days is presented in Table 1. Overall, individuals were more likely (proportion test *p* = .003) to respond “Friday” (*Proportion* = .61 [95% CI .54, .67]) compared to “Monday” (*Proportion* = .39 [.33, .46]).

**Table 1.** Frequencies of responses to temporal ambiguity question cross-classified bystudy day.

	Study day		
Response	Monday	Friday	Total
Monday			
Observed	57	25	82
% within column	47 %	29 %	39 %
Friday			
Observed	64	62	126
% within column	53 %	71 %	61 %

### Generalized linear model

We used R (R Core Team, 2019) and lme4 (version 1.1-21; Bates et al., 2015) to fit generalized linear model (GLM logistic). We entered the answer (Monday = 0 vs. Friday = 1) as the DV, and study day (Monday = 0 vs. Friday = 1) as the factor in a fixed intercept model. The odds ratio (Exp(B)) is reported as an index of effect size.

**Figure 1.**
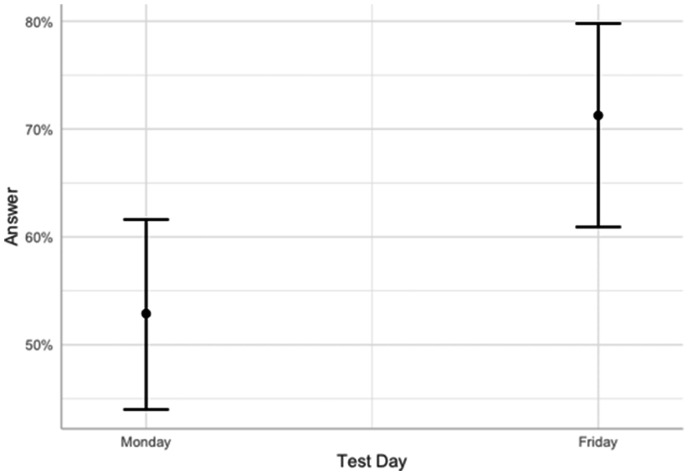
Predicted probabilities of answering “Friday” to temporal ambiguity question as a function of test day. Error bars represent 95% confidence intervals.

There was a study day effect, *estimate* = .79, *SE* = .30, *z value* = 2.65, *p* = .008, such that the odds of answering “Friday” increased when study was administered on Friday (vs. Monday). Specifically, the odds of answering “Friday” in the Friday condition over the odds of answering “Friday” in the Monday condition were *Exp(.79)* = 2.21 [95% CI 1.24, 4.01]. In terms of percent change, the odds of “Friday” answers were 121% higher for the Friday condition vs. Monday condition. Finally, statistical power of the model calculated using the SIMR package (Green & MacLeod, 2016) was .82 at α = .05. Predicted probabilities of “Friday” responses to temporal ambiguity question are presented in Figure 1.

### Proportion tests within conditions

Next, we investigated the probabilities of answering Monday or Friday withing separate conditions (Monday or Friday). Proportion tests indicated that within the Monday condition there was no difference (*p* = .586) in the probability of answering Monday (*Proportion* = .47 [.38, .56]) or Friday (*Proportion* = .53 [.44, .62]). On the other hand, in the Friday condition participants were more likely (*p* < .001) to answer Friday (*Proportion* = .71 [.61, .80]) than Monday (*Proportion* = .29 [.20, .39]).

## Discussion

Our results add to a body of work supporting the notion that the ambiguous temporal language interpretation (at least in English speakers) is contextual and could be related to different non-spatial factors. Specifically, we demonstrated systematic differences in temporal ambiguity resolution as a function of weekdays. Individuals were more likely to respond “Friday” when asked to resolve the temporally ambiguous “Wednesday’s meeting” question on Friday compared to answering the same question on Monday.

In the current study, participants resolving the temporal ambiguity utterance on Friday were more likely to adopt the ME perspective compared to participants in the Monday condition. One possible explanation for this effect is that more distinctive mental representations of Monday and Friday act as temporal primes feeding into decision making about the temporally ambiguous question. Previous research has demonstrated that individuals were faster to retrieve the current day on Monday and Friday compared to other weekdays (Ellis et al., 2015). Thus, Mondays and Fridays seem to be the most saliently represented and distinctive days. This general effect could potentially lead to the adoption of the ME perspective on Fridays, and a shift towards the MT perspective (or at least a truly neutral perspective) on Monday, consistent with the results reported here.^3^

Another possible explanation for the observed effects is that temporal reasoning was influenced by affect. Previous research has demonstrated that individuals associated more negative concepts with Monday and more positive concepts with Friday (Ellis et al., 2015). In addition, there is evidence that mood tends to be more negative on Mondays, and more positive on Fridays (Areni et al, 2011; Ellis et al., 2015; Stone et al., 2012). By this account, individuals would be more likely to adopt the ME perspective on Friday (compared to Monday), again consistent with our results. Of course, the two accounts of the effects of weekdays on temporal ambiguity resolution are not mutually exclusive, and it is possible that a combination of both can be used to explain days of the week effects on the interpretation of the temporally ambiguous question.

The current results suggest that studies using the ambiguous “Wednesday’s meeting” question should interpret their findings with caution, as we demonstrated that the answers to the question fluctuate as a function of the study day. In addition, the question does not seem to be truly neutral, as the overall split suggests a higher rate of Friday responses. Given previously reported effects of various factors such as emotional experiences and personality traits on individuals’ responses to the “Wednesday’s meeting” question, investigating how days of the week interact with other extraneous factors remains an interesting question for future research.

Another outstanding question concerns the relevance of the meeting question for different participants. For example, it is entirely plausible that having a meeting represents an unrealistic scenario for some individuals. This could potentially bias the results.

Finally, it should be noted that the current study addressed a cognitive-linguistic phenomenon rather than a phenomenon of time conceptualization or perception. Clearly, addressing the latter would require investigating analogous temporal effects while controlling for the linguistic effect.

## Conclusion

How do we reason about time? The current study demonstrates that an individual’s resolution of the ambiguous temporal question does not necessarily rely on spatial factors. Our findings raise the interesting possibility that even subtle extraneous factors such as days of the week can influence the resolution of the temporally ambiguous question.

## Supplemental Material

sj-pdf-1-prx-10.1177_0033294120979686 - Supplemental material for The Effects of Day of the Week on Temporal Ambiguity Resolution Click here for additional data file. Supplemental material, sj-pdf-1-prx-10.1177_0033294120979686 for The Effects of Day of the Week on Temporal Ambiguity Resolution by Srdan Medimorec in Psychological Reports

## Notes

1 In addition, our analyses exploratory tested the possibility that participants answering the question on Friday might associate the answer “Friday” with negative affect (i.e., the meeting could be “today”) thus adopting the MT perspective.

2 The materials were presented in a fixed order and no other questionnaires were implemented before the ambiguous temporal question was answered. Some participants proceeded to answer different scales afterwards.

3 It is worth noting that the baseline rate in this study (i.e., the overall average) indicated that individuals were more likely to respond “Friday” than “Monday”. While in the Monday condition participants were at the neutral rate, this still represents a trend towards the MT perspective relative to the baseline.
