# Supplementary material for: The Effects of Day of the Week on Temporal Ambiguity Resolution
Source: Psychol Rep. 2021 Feb 11;125(2):882–9. doi: 10.1177/0033294120979686 (PMC9003772; doi:10.1177/0033294120979686)
Supplement: sj-pdf-1-prx-10.1177_0033294120979686 - Supplemental material for The Effects of Day of the Week on Temporal Ambiguity Resolution [file sj-pdf-1-prx-10.1177_0033294120979686.pdf]

PARTICIPA	TEST_DAY	AGE	GENDER	ANSWER
2	0	21	1	1
3	0	34	1	1
6	1	74	1	1
14	1	35	1	1
16	1	24	1	1
18	1	35	1	1
20	1	28	1	1
22	1	32	1	1
23	1	35	1	1
24	1	22	1	1
25	1	28	1	1
26	1	67	1	1
27	1	28	1	1
29	1	32	1	1
32	1	21	1	1
33	1	33	1	1
34	1	20	1	1
35	1	48	1	1
38	1	40	1	1
40	1	23	1	1
41	1	31	1	1
42	1	33	1	1
43	1	50	1	1
44	1	59	1	1
46	1	33	1	1
47	1		1	1
48	1	19	1	1
49	1	22	1	1
50	1	21	1	1
51	1	30	1	1
52	1	22	1	1
53	1	20	1	1
54	1	31	1	1
55	1	21	1	1
56	1	21	1	1
59	1	25	1	1
60	1	18	1	1
61	1	31	1	1
64	1	28	1	0
66	1	42	1	0
69	1	25	1	0
70	1	36	1	0
71	1	24	1	0
72	1	29	1	0
73	1	20	1	0
78	1	22	1	0

79	1	44	1	0
80	1	19	1	0
81	1	22	1	0
82	1	30	1	0
83	1	21	1	0
87	1	21	1	0
88	1	23	1	0
90	1	18	1	1
91	1	22	1	0
93	0	28	1	1
95	0	45	1	1
96	0	34	1	1
99	0	37	1	1
100	0	40	1	1
102	0	35	1	1
103	0	23	1	1
105	0	41	1	1
108	0	48	1	1
112	0	60	1	1
115	0	43	1	1
116	0	21	1	1
118	0	27	1	1
119	0	23	1	1
120	0	33	1	1
121	0	41	1	1
122	0	23	1	1
123	0	29	1	1
124	0	22	1	1
127	0	26	1	1
128	0	55	1	1
130	0	32	1	1
131	0	42	1	1
133	0	53	1	1
137	0	31	1	1
139	0	44	1	1
140	0	39	1	1
141	0	29	1	1
142	0	19	1	1
143	0	31	1	1
144	0	29	1	1
145	0	28	1	1
146	0	23	1	0
152	0	48	1	0
153	0	47	1	0
154	0	45	1	0
157	0	32	1	0
161	0	30	1	0

162	0	29	1	0
163	0	21	1	0
164	0	24	1	0
165	0	33	1	0
167	0	28	1	0
172	0	47	1	0
173	0	23	1	0
174	0	27	1	0
175	0	32	1	0
176	0	25	1	0
178	0	33	1	0
180	0	45	1	0
181	0	44	1	0
184	0	26	1	0
186	0	42	1	0
189	0	24	1	0
191	0	43	1	0
192	0	20	1	0
196	0	20	1	0
198	0	33	1	0
200	0	39	1	1
201	0	18	1	1
202	0	37	1	1
204	0	52	1	1
208	0	41	1	0
1	0	29	0	1
4	0	22	0	1
5	1	26	0	1
7	1	24	0	1
8	1	51	0	1
9	1	23	0	1
10	1	28	0	1
11	1	32	0	1
12	1	23	0	1
13	1	24	0	1
15	1	58	0	1
17	1	38	0	1
19	1	18	0	1
21	1	30	0	1
28	1	35	0	1
30	1	29	0	1
31	1	25	0	1
36	1	27	0	1
37	1	45	0	1
39	1	24	0	1
45	1	37	0	1
57	1	24	0	1

58	1	23	0	1
62	1	25	0	1
63	1	38	0	0
65	1	22	0	0
67	1	32	0	0
68	1	22	0	0
74	1	27	0	0
75	1	24	0	1
76	1	23	0	1
77	1	53	0	1
84	1	18	0	0
85	1	26	0	0
86	1	21	0	0
89	1	42	0	0
92	0	24	0	1
94	0	19	0	1
97	0	24	0	1
98	0	26	0	1
101	0	21	0	1
104	0	24	0	1
106	0	25	0	1
107	0	51	0	1
109	0	55	0	1
110	0	22	0	1
111	0	43	0	1
113	0	46	0	1
114	0	30	0	1
117	0	52	0	1
125	0	21	0	1
126	0	47	0	1
129	0	34	0	1
132	0	25	0	1
134	0	33	0	1
135	0	26	0	1
136	0	19	0	1
138	0	31	0	1
147	0	25	0	0
148	0	36	0	0
149	0	38	0	0
150	0	35	0	0
151	0	47	0	0
155	0	33	0	0
156	0	29	0	0
158	0	23	0	0
159	0	27	0	0
160	0	53	0	0
166	0	48	0	0

168	0	36	0	0
169	0	35	0	0
170	0	52	0	0
171	0	28	0	0
177	0	27	0	0
179	0	59	0	0
182	0	26	0	0
183	0	36	0	0
185	0	41	0	0
187	0	21	0	0
188	0	51	0	0
190	0	61	0	0
193	0	24	0	0
194	0	63	0	0
195	0	28	0	0
197	0	36	0	0
199	0	23	0	0
203	0	33	0	1
205	0	39	0	1
206	0	45	0	0
207	0	30	0	0
